# Assessing the sustainability of the Nigerian urban reproductive health initiative facility-level programming: longitudinal analysis of service quality

**DOI:** 10.1186/s12913-019-4388-3

**Published:** 2019-08-09

**Authors:** Ilene S. Speizer, Lisa M. Calhoun, Courtney McGuire, Peter M. Lance, Caroline Heller, David K. Guilkey

**Affiliations:** 1 Department of Maternal and Child Health, University of North Carolina Gillings School of Global Public Health, Chapel Hill, NC USA; 2 Carolina Population Center, University of North Carolina at Chapel Hill, Chapel Hill, NC USA; 3 Department of Economics, University of North Carolina at Chapel Hill, Chapel Hill, NC USA

**Keywords:** Sustainability, Nigeria, Family planning, Urban, Quality

## Abstract

**Background:**

To date, there is little information on the sustainability of family planning (FP) service quality after completion of a donor-funded program. This paper examines the sustainability of the Nigerian Urban Reproductive Health Initiative (NURHI) program on quality of FP services in two cities: Ilorin, where the program ended in March 2015 and Kaduna where the program continued.

**Methods:**

Data come from three time periods: 2011, before program implementation; 2014, near Phase 1 completion; and 2017, two-years post Phase 1. In 2011, we undertook a facility audit and provider surveys in all public sector facilities in each city as well as all private facilities mentioned as the source for FP or maternal, newborn, and child health services in a 2010 women’s household survey. In 2014 and 2017, we returned to the same facilities to undertake the facility audit and provider surveys. Quality is measured from principal component analyses of 30 items from the facility audit and provider surveys. Service use outcomes are measured as the ratio of FP clients (total and new) to the number of reproductive health staff members. Multivariate random effect models are estimated to examine changes in the outcomes over time, between NURHI and non-NURHI facilities and by city.

**Results:**

We demonstrate that NURHI facilities had better quality and higher service use than non-NURHI facilities. Further, while quality of services was higher in Ilorin in 2011, by 2014 and three years later (2017), the quality was better in Kaduna where the program continued. In addition, while no difference was found in service utilization between Ilorin and Kaduna in 2014, by 2017, Kaduna had significantly more new FP users than Ilorin.

**Conclusions:**

In Ilorin, quality of services did not continue its strong upward trend after the program ended. Programs need to consider long-term strategies that support continuation of program components post program implementation. This may include ensuring continued training of providers and addressing equipment and commodity stock-outs through system changes rather than specific facility-level changes. The findings from this study can be used to inform future programs seeking to improve quality of FP services in a sustainable manner.

**Electronic supplementary material:**

The online version of this article (10.1186/s12913-019-4388-3) contains supplementary material, which is available to authorized users.

## Background

Increasing modern contraceptive use requires providing quality family planning services, where quality is fairly broadly defined [1, 2]. For instance, per the Family Planning 2020 Initiative, quality includes, among other things, access to a full range of methods, providing clear and medically accurate information to clients, and having providers who are well equipped, technically competent and respectful [3].

To date, much of the literature on quality of care in family planning services in sub-Saharan Africa has focused on cross-sectional or descriptive studies demonstrating the level of quality at a certain point in time and/or comparing quality between the public and private sectors [4–7]. One recent exception by Winston and colleagues [8] examines if program components that sought to increase the quality of services led to increases in service use. To do this the authors use longitudinal facility data from urban areas in Kenya, Nigeria and Senegal. The authors demonstrate that facilities with bigger improvements in service quality had increased service use in the follow-up period [8]. No studies were found that examine whether strategies to improve family planning quality of care sustain following the conclusion of program implementation.

What happens to service quality once a program ends or funding is terminated remains a critical open question. Recently there has been a lively discussion in the literature on the importance of measuring the sustainability of program activities after initial or intensive funding ends [9–11]. Sustainability in these studies focuses on the continuation of program activities or the institutionalization of a program in the long-term [9, 10]. Four main reasons have been proposed to strive for sustainability: to maintain program effects; to capture effects that may only appear following a certain period of time; to reap the benefits of investments; and to prevent the disillusionment of community members and clients, as can happen when programs are discontinued [10]. Reviews of program sustainability, mostly from developed countries, have demonstrated that programs that are more sustainable have certain common characteristics including: a) the program is modifiable; b) there is a program champion to keep the program going; c) there is a good fit between the program and the organization implementing it; d) the benefits of the program are recognized by those implementing it; and e) community-level stakeholders endorse the program [9]. In more recent reviews of program sustainability across program areas and contexts, similar factors that affect whether programs sustain have been identified including: a) characteristics related to the innovation itself (such as the fit, ability to be modified, and its effectiveness or perceived benefit); the organizational context where the program is implemented (climate, culture, and leadership); capacity to continue the program (internal, such as the presence of a champion, and external, such as community involvement); and processes (such as training, collaboration and partnership, and relationship building) [9, 12, 13]. That said, there is evidence that the effect of these factors on sustainability varies by program area [12].

To date, only a few studies focusing on sexual and reproductive health in developing countries have examined program sustainability at the beneficiary or the program level. Studies from Asia on maternal and child health have shown sustainability of use of skilled birth attendants by women [14, 15] and two studies from India examining a family planning program demonstrated continued family planning awareness and use among women exposed to the program after program implementation concluded [16, 17]. More recently, a study from Nigeria demonstrated that those persons exposed to a family planning program were more likely to use family planning post-implementation, but overall exposure declined in the two-years since the program ended [18].

At the program level, sustainability may be measured as continued intervention activities, such as outreach programs or trained providers still practicing their skills or implementing activities after program completion. For example, a project in northwest Tanzania that focused on training village health workers about maternal health showed continuation of the community-supported transport system and community workers providing education and referrals up to five years after the end of the project in communities where these activities were incorporated into the village political systems [19]. A literature review on the sustainability of health interventions in sub-Saharan Africa found 41 studies of sustainability [20]. The main program areas of these studies were HIV/AIDS and malaria and none of the studies specifically focused on family planning programming. The authors found that community ownership and community mobilization, including resource mobilization, were key components of programs that sustained [20].

## Methods

In 2011, a survey of facilities in NURHI project cities (Abuja, Benin City, Ibadan, Ilorin, Kaduna, and Zaria) was undertaken to provide a baseline profile of the family planning (FP) supply environment prior to the launch of the NURHI program. At endline (2014), another survey was undertaken to examine changes in facility level indicators in the same six NURHI study cities. In 2017, a follow-up survey of facilities was undertaken in two cities: Ilorin and Kaduna.^1^ These two study cities were intentionally selected to provide differing perspectives of program activities. In particular, the NURHI program continued in Kaduna in the second phase of NURHI (called NURHI 2) while the program ended in March of 2015 in Ilorin.

The initial set of facilities interviewed in this study was determined at baseline. It included all public sector facilities and all private sector facilities where NURHI was working (or intended to work at baseline) as well as any additional facilities named by female household survey respondents in the baseline household survey from 2010/2011 as locations where they obtained FP or maternal and child health services. At baseline, interviews were undertaken in 72 facilities in Ilorin and 92 in Kaduna. At endline (2014) the project team sought to undertake interviews in the same facilities from baseline. A total of 71 facilities from Ilorin and 83 from Kaduna were included at endline. By the time of the follow-up sustainability study (2017), interviews were undertaken in 70 facilities from Ilorin and 86 facilities from Kaduna. The numbers of facilities change over time primarily due to a small number of refusals at the time of follow-up data collection.

In each study facility at each round of data collection, interviewers implemented a facility audit and provider surveys with up to four providers, depending on the size of the facility. The facility audit interviewed a manager or administrator in the facility and included detailed questions about services offered, number of clients, provider characteristics, quality of services, and equipment in the facility. A copy of the facility audit used in 2017 is included as supplementary materials to this paper (Additional file 1); the tool used in the earlier phases is similar to this tool. Providers were asked questions about their training in FP, services they offer and potential biases by age or marital status with provision of FP. A copy of the provider survey used in 2017 is included as supplementary materials to this paper (Additional file 2); the tool used in the earlier phases is similar to this tool. Facility surveys also included exit interviews with clients obtaining sexual and reproductive health services in a subset of higher volume facilities; exit interview data are not used in this analysis.

Among the facilities included in the study, none were “NURHI supported” facilities at baseline whereas by 2014, NURHI was working in 22 facilities in Ilorin and 22 facilities in Kaduna; this represents 31 and 27% of study facilities in Ilorin and Kaduna, respectively (see Table 1). For the analyses presented below, those facilities identified as “NURHI supported” in 2014 remain in this classification in 2017 for examination of long-term sustainability, even though the NURHI program was no longer working in Ilorin in 2017. We exploit the facility-level data that includes information on the same facilities across multiple time periods, by undertaking multivariate analyses of the quality of services over time, allowing estimation of how quality varies by city, time period, and whether the facility was NURHI supported.

**Table 1:** Characteristics of health facilities by city and time period, MLE facility surveys from Kaduna and Ilorin, Nigeria

	Ilorin			Kaduna		
Variable	Baseline (2011)	Endline (2014)	Sustainability (2017)	Baseline (2011)	Endline (2014)	Sustainability (2017)
NURHI supported facility (%)	0	30.99	31.43	0	26.51	25.58
Managing authority						
Public	40.28	42.25	42.86	31.52	37.35	38.37
Private	59.72	57.75	57.14	68.48	62.65	61.63
Type of facility						
Hospital	75.00	69.01	70.00	70.65	67.47	67.44
Health center	11.11	15.49	15.71	21.74	26.51	26.74
Clinic	5.56	12.68	12.86	2.17	3.61	3.49
Health post/dispensary/other	8.33	2.82	1.43	5.43	2.41	2.33
Number of facilities	72	71	70	92	83	86

The main service delivery measure used in this paper is quality of services. As done in earlier studies [4, 8, 21], we create a quality index to measure the multiple, correlated components of service quality. The quality index is created from principal components analysis (PCA) of the facility data from the three time periods in one PCA using the first component of the score. This component captures about 22% of the total variance for the 30 items that make up the measure. The quality index incorporates the following measures: method availability (10 methods are captured); stockout of each method at the facility (8 methods); information, education and communications (IEC) materials available (8 types of IEC materials measured); outreach activities (3 types of activities); and the mean number of providers trained in the last year (see Table 2 for a description of these variables). We create an overall quality index which is examined by city and time period. This quality index is used as an outcome variable as well as a key independent variable for the analysis of FP service use. In addition, related to each of the components of quality (method availability, stockouts, IEC, outreach, and provider training), we create indexes for more detailed analyses. Notably, except for training, we use PCA to create the component indexes. Training is calculated as the average number of providers trained.

**Table 2:** Inputs for quality measures and service delivery outcomes included in the facility surveys

Variable	Measurement approach
Method availability index	
Are each of the following methods currently available?	Facility audit – Y/N
IUD	
Implant	
Injectable	
Combined oral contraceptive pill	
Progesterone only pill	
Emergency contraception	
Male condom	
Female condom	
Female sterilization	
Male sterilization	
Are the following information, education and communication (IEC) items observed?	Facility audit – Y/N
Posters	
Flip Chart	
Brochure	
Info sheet	
Job Aid	
Demo	
Counsel Card	
FP Samples	
Has the facility had a stockout of the following methods in the last 30 days?	Facility audit – Y/N
IUD	
Implant	
Injectable	
Combined oral contraceptive pill	
Progesterone only pill	
Emergency contraception	
Male condom	
Female condom	
Does the facility have the following outreach activities?	Facility audit – Y/N
Outreach with Information, Education and Communication	
Outreach program that discusses family planning	
Does health talks on family planning to the community	
Provider training – average number trained	Provider interviews
Number of new acceptors of family planning	Facility audit (12 months)
Number of family planning users (including new acceptors)	Facility audit (12 months)
Number of reproductive health staff members	Facility audit

Two FP service use measures are used as outcomes as well: the ratio of the number of new FP clients in the last year to the number of FP and reproductive health (RH) staff, and the ratio of the total number of FP clients in the last year to the number of FP and RH staff. The ratio adjusts for the fact that larger facilities have more staff and will have more clients. The explanatory variables in the model are all dummy indicator variables: indicators for survey round, city, NURHI supported facility, and whether the facility was private. In addition, interactions are examined between time point (e.g., 2014 and 2017) and the city and whether the facility was a NURHI facility. Three clinics were dropped for the new client analyses and four from the total client analyses because they had extremely high and unrealistic values for the dependent variables and had an undue influence on the regression results.

Descriptive analyses are used to examine the characteristics of the study facilities at each time point. In addition, we examine the overall quality measure as well as the components of quality and service use across the two cities and at each time point. Because the data are longitudinal at the facility level, we used longitudinal regression methods. In particular, we specified a random effects model and used maximum likelihood estimation methods. Since the model is linear, the coefficient estimates from these models are also the marginal effects; all multivariate results are presented with the marginal effects and their 95% confidence intervals. Random effects estimation estimates the standard deviation of the two components of the error term in addition to the regression coefficients. The standard deviation of the time invariant error is labeled “Sigma mu” and the standard deviation of the time varying error is labeled “Sigma epsilon” in the tables. In all instances except one, both error components were highly significant indicating the importance of correcting for both sources of error. However, also in all cases, the time varying error component was considerably larger than the time invariant component indicating that time varying unobservables were relatively more important factors. Another assumption for the random effects model is that the explanatory variables are not correlated with the time invariant error. We tested this null hypothesis using a robust version of the standard Hausman test (see, for example. Wooldridge, 2010, page 132 [22]). In all cases, we failed to reject the null hypothesis which again is supportive of the validity of our estimation strategy. All multivariate analyses were performed using the *xtreg* program from the Stata statistical software package and the robust version of the Hausman test used the user created *xtoverid* Stata add on.

## Results

Table 1 presents the characteristics of the facilities included in the study. As mentioned earlier, no facilities were classified as NURHI facilities at baseline since the program had not yet begun at that time. By the time of endline data collection (2014), 31 and 26.5% of study facilities in, respectively, Ilorin and Kaduna were NURHI facilities. Table 1 also shows that about two-fifths of study facilities in Ilorin were public sector facilities while only about a third of facilities in Kaduna were public sector facilities. In both cities, most of the facilities included were hospitals. The next most common type of facility was health centers followed by clinics.

Table 3 presents the results of the principal component analyses of the overall quality index as well as for the component indexes of the overall quality (method provision index, IEC index, stock out index, and outreach index) and the average number of providers interviewed that were trained by city and time period. Because these measures are from principal components analysis, the interpretation is not straightforward, so we focus on the signs and trends. The overall quality index in both cities was lowest at baseline (2011). By endline (2014), the overall quality improved in both cities. By the sustainability study (2017), overall quality remained positive and the values were larger than in 2014 in both cities. Availability of methods in the facilities over time and by city has a similar pattern to that of the overall quality – it was negative and increased over the follow-up periods. Stockouts are not considered good and thus the sign of the index is in the opposite direction such that there were more stockouts in Ilorin at baseline, these reduced by endline but increased again by 2017. For Kaduna, there were fewer stockouts between baseline and endline and stockouts increased by 2017 as well. The outreach index improved in both cities between baseline and endline but declined somewhat between endline and 2017. Finally, provider training in Ilorin increased between baseline and endline but declined again by 2017 whereas in Kaduna, where the program continued, provider training increases across the three time periods.

**Table 3:** Description of service outcomes at baseline (2011), endline (2014), and sustainability study (2017) in the analysis sample by city, MLE facility surveys from Kaduna and Ilorin, Nigeria

	Ilorin			Kaduna		
Variable	Baseline (2011)	Endline (2014)	Sustainability (2017)	Baseline (2011)	Endline (2014)	Sustainability (2017)
Overall quality index (mean)	−0.842	0.284	0.686	−1.758	0.734	1.167
Method provision index (mean)	−0.038	0.262	0.561	−1.318	−0.324	0.940
IEC materials index (mean)	−0.861	−0.091	0.478	−1.019	0.983	0.736
Stock out within 30 days index (mean)	0.343	−0.385	0.121	−0.057	− 0.442	0.291
Outreach index	0.044	0.222	0.196	−0.571	0.134	0.196
Mean number of providers trained on FP in the last one year	0.053	0.275	0.080	0.063	0.163	0.291
Mean (median) number of new acceptors per reproductive health staff member in the last 12 months^a^	13.70 (6.00)	22.75 (14.92)	18.95 (9.02)	10.40 (4.75)	43.55 (16.42)	42.90 (21.11)
Mean (median) number of FP users per reproductive health staff member in the last 12 months^a^	30.02 (12.07)	49.87 (37.50)	50.77 (21.11)	36.80 (12)	50.58 (21.23)	96.19 (37.83)
Total number of facilities	72	71	70	92	83	86

All indexes calculated using principal components analysis. ^a^Sample size slightly smaller due to missing data from some facilities on number of users

Also shown in Table 3 are the service use outcomes measured as the number of new FP acceptors in the last 12 months per reproductive health staff member and the total number of family planning users in the last 12 months per reproductive health staff member. In Ilorin, both service delivery outcomes increase between baseline and endline and then decline somewhat by 2017 whereas in Kaduna, both outcomes continue to increase over time with large increases in total FP users per staff member between endline and 2017.

Multivariate random effects models were estimated to examine whether the quality of services is related to NURHI program support and if there were changes in quality over time and across the two cities. These results are presented in Table 4 (overall quality index) and Table 5 (components of quality). Table 4 demonstrates that NURHI supported facilities were of higher quality than non-NURHI supported facilities (marginal effect: 2.32; 95% CI: 1.58; 3.06; *p* < 0.001). In 2011, Ilorin facilities were of higher quality than Kaduna facilities (the Ilorin marginal effect is positive and significant), however, the interaction between Ilorin and the two time periods (2014 and 2017) indicates that by 2014, Ilorin facilities had lower overall quality than Kaduna facilities and this continues into 2017. That said, over time in both cities the quality of services is significantly better than at baseline (positive and significant marginal effects on the 2014 and 2017 variables). There is no difference in NURHI facilities in 2017 as compared to the earlier time period (2014). Finally, private facilities have lower quality across all time periods and cities.

**Table 4:** Random effects results predicting quality of services in facilities, MLE facility surveys from Kaduna and Ilorin, Nigeria

	Overall Quality Index	
	Marginal	95% CI
Ilorin (ref: Kaduna)	0.77	0.14; 1.40*
Survey wave		
Baseline – 2011 (ref)	–	
Endline – 2014	1.76	1.19; 2.33***
Sustainability – 2017	2.34	1.78; 2.90***
NURHI- supported facility (ref: not NURHI)	2.32	1.58; 3.06***
Private facility (ref: public)	−1.85	−2.34; − 1.36***
Ilorin and Endline (2014) interaction	−1.39	−2.19; −0.59***
Ilorin and sustainability (2017) interaction	−1.42	−2.22; − 0.62***
NURHI facility and sustainability (2017) interaction	−0.57	− 1.46; 0.31
Error components:		
Sigma mu	0.96	0.74; 1.24***
Sigma epsilon	1.79	1.66; 1.94***
Number of observations	474	
Number of unique facilities	172	

Note: overall quality index created from PCA of the indices for the components of quality

*p ≤ 0.05; ***p ≤ 0.001

**Table 5:** Random effects results predicting program components scores among facilities, MLE facility surveys from Kaduna and Ilorin, Nigeria

	Training		Outreach		IEC materials		Method provision		Stock-out in last 30 days	
	Marginal	95% CI	Marginal	95% CI	Marginal	95% CI	Marginal	95% CI	Marginal	95% CI
Ilorin (ref: Kaduna)	−0.02	− 0.09; 0.05	0.49	0.07; 0.91*	0.07	−0.50; 0.64	1.25	0.77; 1.72***	0.38	−0.09; 0.85
Survey wave										
Baseline – 2011 (ref)	–		–		–		–		–	
Endline – 2014	0.09	0.02; 0.15**	0.52	0.12; 0.92*	1.48	0.94; 2.02***	0.59	0.16; 1.02**	−0.35	−0.80; 0.09
Sustainability – 2017	0.20	0.14; 0.26***	0.54	0.15; 0.94**	1.26	0.73; 1.80***	2.09	1.66; 2.52***	0.51	0.07; 0.94*
NURHI- supported facility (ref: not NURHI)	0.03	−0.05; 0.11	0.40	−0.10; 0.90	1.67	0.99; 2.35***	1.37	0.81; 1.93***	−0.22	−0.78; 0.33
Private facility (ref: public)	−0.06	−0.11; − 0.01*	−1.48	−1.79; −1.17***	−1.17	−1.59; 0.75***	−0.45	− 0.82; − 0.07*	−0.14	− 0.48; 0.21
Ilorin and Endline (2014) interaction	0.12	0.03; 0.21**	−0.49	−1.06; 0.07	−1.26	−2.02; − 0.49***	−0.71	−1.32; − 0.11*	−0.30	− 0.93; 0.32
Ilorin and sustainability (2017) interaction	−0.20	− 0.29; − 0.11***	−0.50	− 1.06; 0.07	−0.46	−1.22; 0.30	− 1.66	−2.27; − 1.06***	−0.53	−1.15; 0.10
NURHI facility and sustainability (2017) interaction	0.05	−0.05; 0.15	−0.18	− 0.81; 0.45	−0.07	− 0.92; 0.78	−0.88	−1.56; − 0.20*	−0.44	− 1.13; 0.26
Sigma mu	0.09	0.06; 0.12***	0.47	0.30; 0.72***	0.69	0.47; 1.00***	0.75	0.57; 0.97***	0.55	0.37; 0.82***
Sigma epsilon	0.20	0.19; 0.22***	1.27	1.18; 1.38***	1.71	1.59; 1.86***	1.36	1.26; 1.48***	1.41	1.30; 1.52***
Number of observations	474		474		474	474			474	
Number of unique facilities	172		172		172	172			172	

Note: each outcome created based on PCA of component parts, except for training which is just the average number of providers trained. *p ≤ 0.05; **p ≤ 0.01; ***p ≤ 0.001

Table 5 presents similar models for each of the component indexes from the overall quality index. The results for the method provision index are the same as those presented for overall quality – that is NURHI facilities perform better than non-NURHI facilities, Ilorin had better method provision at baseline but it is not better than Kaduna by endline and 2017, and overall, method provision improved over time. For the training index, Ilorin and Kaduna were similar at baseline and by endline, Ilorin was doing better than Kaduna. That said, by 2017, Kaduna was doing better on provider training than Ilorin (marginal effect: -0.20; 95% CI: − 0.29; − 0.11; *p* < 0.001). Improvements in availability of IEC materials are observed over time and NURHI facilities do better than non-NURHI facilities. In addition, Kaduna facilities in 2014 have better IEC than Ilorin facilities but there is no difference by 2017. For outreach, Ilorin was doing better at baseline and while improvements happened over time, by endline (2014) and 2017, Ilorin is doing worse on outreach (*p* < 0.10). Further, private facilities are doing worse than public facilities on outreach. The last index shown in Table 4 is stockouts. There are fewer factors related to stockouts and stockouts are found to increase by 2017 relative to 2011. This likely reflects state-level rather than city-level commodity distribution systems that are not as controlled by specific facilities and the NURHI program.

Table 6 presents the multivariate random effects models of the effect of quality, city and NURHI facilities on the total number of family planning users in the last year per reproductive health staff member. Two models are shown, the first using the overall quality index and the second using the individual component indexes of quality. Both models control for the facility and time characteristics shown previously to be related to the quality of services. Model 1 shows that higher quality facilities have a higher ratio of clients to providers, although the effects do not attain significance (*p* = 0.120). Between baseline and endline, there is not a significant increase in the number of family planning clients to providers; however, by the time of the sustainability study (2017), all facilities generally had more clients to providers (*p* < 0.001) and NURHI facilities had more clients to providers than non-NURHI facilities (*p* = 0.009) (and private facilities had fewer – *p* < 0.001). The interaction between time and Ilorin was only marginally significant in 2017 such that there was no difference in service delivery between the two cities in 2011 and 2014 when the program ended. However, by 2017, Kaduna facilities had a slightly higher client load (*p* = 0.081). The results of Model 2 demonstrate that those facilities with more methods available have more family planning clients to providers and those with fewer stockouts also have more clients to providers. All the control variables perform similarly in Model 2 with the exception of the Ilorin and 2017 interaction which is not significant.

**Table 6:** Random effect model results predicting the effect of overall quality and program component scores on the number of FP users in the last year per reproductive health staff member, MLE facility surveys from Kaduna and Ilorin, Nigeria

	Number of FP users per reproductive health staff member			
	Model 1		Model 2	
	Marginal	95% CI	Marginal	95% CI
Overall quality index	2.57	−0.67; 5.81	na	
Training index	Na		17.71	−9.90; 45.32
Method provision index	Na		5.43	0.27; 10.60*
Stock-out (30 days) index	Na		−5.90	−10.10; − 1.70**
IEC index	Na		−0.95	−4.48; 2.57
Outreach index	Na		−0.94	−5.48; 3.61
Ilorin (ref: Kaduna)	−9.29	−32.55; 13.97	−12.34	− 36.12; 11.44
Survey wave				
Baseline – 2011 (ref)	Ref		Ref	
Endline – 2014	0.70	−20.75; 22.15	−0.13	−21.52; 21.26
Sustainability – 2017	34.63	13.72; 55.54***	31.08	8.69; 53.46**
NURHI- supported facility (ref: not NURHI)	33.87	8.61; 59.13**	32.54	7.60; 57.50*
Private facility (ref: public)	−36.61	−53.30; −19.91***	−41.57	−58.64; 24.50***
Ilorin and Endline (2014) interaction	7.95	−22.08; 37.98	4.47	−25.93; 34.86
Ilorin and sustainability (2017) interaction	−26.30	−55.87; 3.27	−20.01	−50.80; 10.79
NURHI facility and sustainability (2017) interaction	2.94	−27.16; 33.04	1.83	−28.42; 32.09
Error Components:				
Sigma mu	24.59	16.68; 32.27***	22.40	14.23; 35.27***
Sigma epsilon	59.97	55.13; 65.24***	59.84	54.99; 65.13***
Number of observations	424		424	
Number of unique facilities	170		170	

na – not applicable for specific model; excludes outliers. *p ≤ 0.05; ** *p* ≤ 0.01; *** *p* ≤ 0.001

Table 7 presents the multivariate random effect results for the outcome of new family planning clients per reproductive health staff member. These results are like those presented in the previous table such that quality is positive but does not attain significance (*p* = 0.196) and the number of new clients to providers is significantly higher over time and in NURHI-supported facilities. In addition, by 2017, the number of new clients to providers is lower in Ilorin as compared to Kaduna (Ilorin and 2017 interaction – marginal effect: -20.57; 95% CI: − 35.42; − 5.72; *p* = 0.007). Using the specific quality components, we find that method availability (*p* < 0.10) and stockouts remain important in this model in the same direction as earlier.

**Table 7:** Random effect model results predicting the effect of overall quality and program component scores on the number of new family planning acceptors in the last year per reproductive health staff member, MLE facility surveys from Kaduna and Ilorin, Nigeria

	Number of new acceptors per reproductive health staff member			
	Model 1		Model 2	
	Marginal	95% CI	Marginal	95% CI
Overall quality index	1.06	−0.55; 2.67	na	
Training index	na		5.32	−8.06; 18.70
Method provision index	na		2.24	−0.30; 4.78
Stock-out (30 days) index	na		−2.61	−4.70; −0.52*
IEC index	na		0.15	−1.60; 1.89
Outreach index	na		−1.10	−3.33; 1.13
Ilorin (ref: Kaduna)	0.71	−10.82; 12.23	−0.18	−11.98; 11.62
Survey wave				
Baseline – 2011 (ref)	Ref		ref	
Endline – 2014	10.81	−0.11; 21.73	10.15	−0.93; 21.24
Sustainability – 2017	19.45	8.83; 30.08***	18.05	6.54; 29.55**
NURHI- supported facility (ref: not NURHI)	16.88	4.28; 29.49**	14.69	2.39; 26.98*
Private facility (ref: public)	−11.34	−19.35; −3.33**	−14.48	−22.37; −6.59***
Ilorin and Endline (2014) interaction	−7.42	−22.58; 7.73	−8.35	−24.08; 7.38
Ilorin and sustainability (2017) interaction	−20.57	−35.42; −5.72**	−18.54	−34.36; − 2.71*
NURHI facility and sustainability (2017) interaction	1.81	−13.22; 16.83	1.61	−13.99; 17.20
Error Components:				
Sigma mu	9.66	5.35; 17.43***	3.24	+
Sigma epsilon	29.88	27.42; 32.56***	30.90	28.84; 33.10***
Number of observations		416	416	
Number of unique facilities		168	168	

na – not applicable for specific model; excludes outliers. + The maximum likelihood estimator did not report a standard error in this case indicating a large standard error and an insignificant effect for the time invariant error. * *p* ≤ 0.05; ** *p* ≤ 0.01; ***p ≤ 0.001

## Discussion

This study seeks to determine the post-program sustainability of the quality of FP services by comparing a city where a program continued to a city where the it ended two years earlier. Determining the level and extent of sustainability is important for informing future programs seeking to improve the quality of FP services in the short and long term.

Our results demonstrate that NURHI facilities tended to have better quality and higher service use than non-NURHI facilities. Further, while quality of services was higher in Ilorin at baseline (2011), by endline (2014) and three years later (2017), the quality was better in Kaduna where the program continued. This was shown for the overall quality index as well as the components of training, outreach, and method availability; each of these were lower in 2017 in Ilorin as compared to Kaduna. In addition, while no difference was found in service utilization between Ilorin and Kaduna at endline, by 2017 Kaduna had significantly more new users than Ilorin. These findings demonstrate that quality of services was maintained in NURHI facilities from Ilorin after the program ended. Further, while quality of services increased in 2014 and 2017 in both cities, quality did not continue to improve at the same pace in Ilorin as it did in Kaduna where the program continued. Two years after program completion, there were fewer new FP clients in the Ilorin facilities.

The literature on sustainability points to reasons why we care about sustainability of program activities including reaping the benefits of investments made and determining longer-term effects that may be missed when measurement ends at program completion [11]. Further, program activities may diffuse or spread, for example by trained providers training others, by outreach workers continuing to give messages after the program ends and spreading messages to locations beyond their normal catchment area, or by friendly competition whereby NURHI improved facilities lead other facilities to improve their service quality. Without an explicit measurement strategy to capture what happens post-implementation, this information goes unknown and cannot be used for design of future programs that are sustainable and scalable.

Our findings are not without limitations. First, there is no standardized measurement of quality of FP services [5] and therefore we used proxy measures based on a facility audit and provider interviews. Second, information was not available from provider client observations and mystery clients to strengthen the measurement of program quality at each time period. Further, while exit interviews were undertaken as part of the study and can often give information on client satisfaction, these were only undertaken in high-volume facilities, limiting their utility for this study. Third, we were not able to examine directly whether quality of services affects reported contraceptive use since linking women from household surveys to facilities is complex, particularly in urban settings [23]. That said, we did show that facility quality is related to service use, measured as the ratio of family planning clients (or new clients) to number of reproductive health staff members. Fourth, the sample of facilities was too small to create all the potentially relevant interactions including the three-way interaction of being a NURHI facility in Ilorin in 2017. Qualitative data would be useful for better understanding detailed changes across various facilities (NURHI and non-NURHI) by 2017 in both Kaduna and Ilorin.

## Conclusions

This study demonstrates that the quality of services in the city where the program was terminated did not maintain its upward trend from the earlier period. On the other hand, service quality continued to improve in the city where the program continued. When designing new programs, WHO and ExpandNet recommend “beginning with the end in mind” [24]. This seems particularly relevant for programs that have a short time duration and are seeking to improve quality of services. Identifying long-term strategies that support continuation of the key components of the program is essential. For example, programs need to ensure that a system is developed to address stockouts beyond the finite program timeline; identify strategies for continued training of providers since there is significant provider turn-over in many of these settings; and develop an approach that ensures that facility improvements are maintained including having a strategy to maintain improvement and repair equipment post-program. These types of approaches should lead to sustained quality improvements beyond the life of the program.

## Additional files

Additional file 1: Nigeria Sustainability SDP Service Provider Survey.pdf – Provider survey used as part of this study. (PDF 1065 kb)

Additional file 2: Nigeria Sustainability SDP Facility Audit.pdf – Facility audit used as part of this study. (PDF 1145 kb)

## Data Availability

The dataset supporting the conclusions of this article is available from the corresponding author and can be requested at this site: https://dataverse.unc.edu/dataverse/mle

## Abbreviations

BMGF: Bill & melinda gates foundation
CCP: Center for communication programs
FP: Family planning
IEC: Information, education, and communication
MLE: Measurement, Learning & Evaluation project
NURHI: Nigerian urban reproductive health initiative
PCA: Principal components analysis
RH: Reproductive health

## Footnotes

1 Note that in 2017, facility-level data were also collected in a third city, Jos, where NURHI program activities were never undertaken; data from Jos are not presented in this paper.
