# Supplementary material for: Assessing the sustainability of the Nigerian urban reproductive health initiative facility-level programming: longitudinal analysis of service quality
Source: BMC Health Serv Res. 2019 Aug 9;19:559. doi: 10.1186/s12913-019-4388-3 (PMC6688378; doi:10.1186/s12913-019-4388-3)
Supplement: Supplementary file 1 — Nigeria Sustainability SDP Service Provider Survey.pdf – Provider survey used as part of this study. (PDF 1065 kb) [file 12913_2019_4388_MOESM1_ESM.pdf]

QUESTIONNAIRE IDENTIFICATION NO:[__|__|__|__|__|__|__|__]
5 digit facility code + 3 digit prov code

MEASUREMENT, LEARNING & EVALUATION PROJECT
FOR THE URBAN REPRODUCTIVE HEALTH INITIATIVE

Measurement, Learning & Evaluation (MLE) Project
Service Provider – Nigeria – 2017

IDENTIFICATION

CITY NAME & CODE ________________________________ (Ilorin=4, Kaduna=5, Jos=9)	[__]
LGA NAME & CODE________________________________	[__|__|__]
LOCALITY NAME & CODE ________________________________	[__|__|__|__]
FACILITY NAME AND CODE ________________________________	[__|__|__|__|__]
PROVIDER NAME AND CODE (FROM THE FACILITY AUDIT LIST – Q10d)____________________	[__|__|__]

RESPONDENT: NOT INTERVIEWED = 1 PREVIOUSLY INTERVIEWED AT ANOTHER FACILITY = 2 (END) [ ]

IF PREVIOUSLY INTERVIEWED, OTHER FACILITY NAME AND CODE________________________ [| | | |]

INTERVIEWER VISITS

**VISIT No.**	**1**	**2**	**3**	**FINAL VISIT**
DATE	DAY/ MONTH/YEAR [____/_____/_**17**_]	DAY/ MONTH/ YEAR [____/_____/__**17**_]	DAY/ MONTH/ YEAR [____/_____/__**17**_]	DAY [____|____]<br>MONTH [____|____]<br>YEAR [_**2**_|_**0**_|_**1**_|_**7**_]
INTERVIEWER'S NAME	______________	______________	______________	______________
INTERVIEWER CODE	[		]	[		]	[		]	[		]
RESULT*	[ ]	[ ]	[ ]	[ ]
**NEXT VISIT:**<br>**DATE:**	[___/_____/17_]	[___/_____/17_]	[___/_____/17_]	TOTAL NO. OF VISITS
**TIME:**	[	] [	]<br>H H M M	[	] [	]<br>H H M M	[	] [	]<br>H H M M	[ ]

***RESULT CODES:**
1. COMPLETED
2. RESPONDENT NOT AVAILABLE
3. POSTPONED
4. REFUSED
5. PARTLY COMPLETED
6. OTHER __________________________ (Specify)

SUPERVISOR	OFFICE EDITOR	KEYED BY
NAME ______________________	NAME ______________________	NAME ______________________
CODE [____|____]	CODE [____|____]	CODE [____|____]
DATE [____/_____/_**17**_]<br>DD MM YY	DATE [____/_____/_**17**_]<br>DD MM YY	DATE [____/_____/_**17**_]<br>DD MM YY

QUESTIONNAIRE IDENTIFICATION NO:[__|__|__|__|__|__|__|__]
5 digit facility code + 3 digit prov code

IDENTIFICATION

BACKGROUND INFORMATION

Source	Questions	Coding	Skip
Q1.	RECORD THE TIME<br><br>(IN 24 HOUR FORMAT)	Hour ………… [ ][ ] Minutes …….. [ ][ ]	
Q2.	SEX OF PROVIDER INTERVIEWED	MALE…………………………………1<br>FEMALE………………………….……..2	
Q3.	How long have you been working here at this facility?	YEARS… [ ][ ]<br><br>LESS THAN ONE YEAR =00<br>DON'T KNOW = 98	
Q4.	What cadre of staff are you?	OBSTETRICIAN/GYNECOLOGIST……………..…………..01<br>GENERAL SURGEON…………………………….....…..……02<br>PEDIATRICIAN…………………………………………..……..03<br>GENERAL PHYSICIAN………………………………………..04<br>THEATRE NURSE………………………………………..……05<br>NURSE/MIDWIFE………………………………………..….06<br>NURSE…………………………………………………….......07<br>MIDWIFE…………………………….....……………………08<br>COMMUNITY HEALTH EXTENSION WORKER (CHEW)…09<br>COMMUNITY HEALTH OFFICER (CHO)……………………10<br>VCT COUNSELOR……………………………………………..11<br>OTHER ____96<br>(SPECIFY)	
Q5.	How old were you at your last birthday?	YEARS…….. [ ][ ]	
Q6.	What is your religion?	CHRISTIAN-CATHOLIC………………………………………1<br>CHRISTIAN-PROTESTANT/OTHER CHRISTIAN…………2<br>ISLAM…………………………...……………………………3<br>TRADITIONAL…………………………………………………4<br>NO RELIGION ……………….………………………...…5<br><br>OTHER____6<br>(SPECIFY)	
Q7.	In which department or unit do you work?	GENERAL OUTPATIENT DEPARTMENT (GOPD) ... 01<br>OBSTETRICS AND GYNECOLOGY …..……………… .02<br>SURGERY ……..……………………………...………..03<br>PEDIATRICS ……..…………………………………….04<br>FAMILY PLANNING DEPARTMENT …..………………05<br>INFANT AND CHILD CARE ……………………………..06<br>ANC…………………………………………………..…07<br>HIV TESTING OR STI/HIV TREATMENT……………..08<br>Other ____96<br>(SPECIFY)	
Q7a	Has your salary payment ever been delayed by more than one week?	YES…………………………………1<br>NO…………………………………..2	
Q7b	How many times in the last year has your salary payment been delayed?	NUMBER OF DELAYED PAYMENTS [ ][ ]<br>EVERY PAY PERIOD…………………………………………00<br>NEVER…………………………………………………………97	
Q7c	When was the last time your salary payment was delayed?	WITHIN THE PAST MONTH……………..1<br>WITHIN THE PAST 2-6 MONTHS………2<br>WITHIN THE PAST 7-11 MONTHS…………………3<br>WITHIN THE PAST 1- 3 YEARS…………….4<br>WITHIN THE PAST 4-5 YEARS…………….5<br>MORE THAN 5 YEARS AGO………………6<br>DON'T REMEMBER………………………………..8<br>NEVER…………………………………………………………97	
Q8.	How many years have you been working as a health care provider?	NUMBER OF YEARS: [ ][ ]	

QUESTIONNAIRE IDENTIFICATION NO:[__|__|__|__|__|__|__|__]
5 digit facility code + 3 digit prov code

IDENTIFICATION			
Q9.	How many years ago did you finish your **pre-service** training?	YEARS AGO….. [|]<br>LESS THAN ONE YEAR = 00<br>NO PRE-SERVICE TRAINING=97	

Q10.	Have you received any **in-service** training on family planning?	YES…………………………………1<br>NO…………………………………..2 →	**Q13a**
Q11.	Who provided this training?<br><br>**MULTIPLE RESPONSES POSSIBLE.**<br><br>**CIRCLE ALL MENTIONED.**	NURHI/FPPN ……………………………….. ..A<br>UNFPA ………………………………………….B<br>WHO ……………………………………………C<br>SFH ……………………………………………..D<br>JPIEGO …………………………………………E<br>MARIE STOPES ………………………………F<br>PATH ……………………………………………G<br>PPFN ……………………………………………H<br>STATE/ MOH ……………………………………I<br>ISHIP ………………………………………………J<br>OTHER ____________________________ X<br>(SPECIFY)	
Q12.	What types of in-service trainings did you receive?<br><br>PROBE – Anything else?<br><br>**MULTIPLE RESPONSES POSSIBLE.**<br><br>**CIRCLE ALL MENTIONED.**	FP INTERPERSONAL COMMUNICATION AND COUNSELLING………………………… ………..A<br>INITIAL FP TRAININGS ……………………………… .. B<br>REFRESHER FP TRAINING ……………………………C<br>LONG-ACTING AND PERMANENT FP METHODS... .D<br>TRAINING OF TRAINERS OF FP PROVIDERS ……. E<br>CONTRACEPTIVE LOGISTICS AND MANAGEMENT SYSTEM (CLMS) TRAINING …………F<br>SUPERVISORY SKILLS TRAINING…………………… G<br><br>OTHER ___________________________________ X<br>(SPECIFY)	
Q13.	How long ago was the last **in-service** family planning training that you attended?	DAYS AGO…………………..1 [|]<br>WEEKS AGO………………..2 [|]<br>MONTHS AGO…................3 [|]<br>YEARS AGO…....................4 [|]<br>DON'T REMEMBER………998	
Q13a	Has this facility ever participated in a Whole Site Orientation?	YES……………………………………1<br>NO……………………………………..2 →<br>DON'T KNOW………………………..8 →	**Q14**<br>**Q14**
Q13b	When was the last Whole Site Orientation?	2017…………..………………………..1<br>2016……………………………………2<br>2015……………………………………3<br>2014……………………………………4<br>2013……………………………………5<br>2012……………………………………6<br>BEFORE 2012………………………..7<br>DON'T KNOW………………………..8	
Q13c	Which organization coordinated the Whole Site Orientation?<br><br>MULTIPLE RESPONSES POSSIBE	NURHI. . . . .A<br>MOH .B<br>MARIE STOPES . . . . .C<br>PPFN………………………D<br>SFH…………………………..E.<br>OTHER ________________X	
Q14.	Are you a member of the NURHI supported Family Planning Provider Network (FPPN)?	YES……………………………………………………….1<br>NO………………………………………………………..2 →	**Q22**
Q15.	Have you attended any of the FPPN meetings?	YES……………………………………………………..1<br>NO……………………………………………………….2<br>DON'T KNOW………………………………………….8	

Q19.	Do you discuss family planning with other providers <u>who are NOT FPPN members</u>?	YES……………………………………………………1 →<br>NO……………………………………………………. ….2 →<br>DON'T KNOW NON-FPPN PROVIDERS …………8 →	**Q22a**<br>**Q22a**<br>**Q22a**
Q22.	Have you interacted with providers <u>who are members of the FPPN</u>?	YES……………………………………………………1<br>NO……………………………………………………...2<br>DON'T KNOW/DON'T KNOW FPPN MEMBERS ….8	
Q22a	Have you participated in the quarterly Facility Committee Meetings/ NURHI QIT?	YES . . . . 1<br>NO 2	
Q22b	Have you ever connected with the NURHI WhatsApp FP forum?	YES . . . . 1<br>NO . . . . 2 →	**Q22d**
Q22c	When was the last time you connected with the NURHI WhatsApp FP forum?	DAYS AGO……………………1<br>WEEKS AGO………………..2 [__|__]<br>MONTHS AGO…...............3 [__|__]<br>YEARS AGO…....................4 [__|__]<br>DON'T REMEMBER/DON'T KNOW………998	
Q22d	Have you ever done community mobilization activities with a NURHI social mobilizer?	YES . . . . 1<br>NO . . . . 2<br>DON'T KNOW………………8	
Q22e	Have you ever traveled outside your usual duty station with a NURHI social mobilizer to provide clinical services?	YES . . . . 1<br>NO 2	
Q22f	In the last year, have you made a family planning health referral in the community while working alongside a NURHI social mobilizer?	YES . . . . 1<br>NO 2	
Q22g	Do you have the NURHI movie downloaded on your smartphone or on a CD?<br><br>CIRCLE ALL THAT APPLY	YES, APP OBSERVED………………………………A<br>YES, APP REPORTED, NOT SEEN…………….B<br>YES, CD OBSERVED…………………………………C<br>YES, CD REPORTED, NOT SEEN………………D<br>NO, HAS NEITHER APP NOR CD……………..Y →<br>DON'T KNOW…………………………………………Z →	 <br><br><br>**Q28**<br>**Q28**
Q22h	How often do you watch the NURHI movie by app or CD?	EVERY DAY . . . . 1<br>WEEKLY . . . . . ……... . . . . . . 2<br>MONTHLY……………………..3<br>QUARTERLY…………………4<br>NEVER………………………..5<br>OTHER________________6<br>DON'T KNOW…………………8	

TRAINING ON FAMILY PLANNING

Now, I will ask you few questions related to training on FP.

Q28. CHECK Q09 AND Q10 ON PRE-SERVICE AND IN-SERVICE TRAINING:

HAS HAD BOTH PRE AND IN-SERVICE TRAINING ☐ ↓
(Q9=00 OR HIGHER AND Q10=1)
<u>THEN ANSWER Q28a-Q28d</u>

HAS HAD IN-SERVICE TRAINING ONLY ☐ → **Q28b**
(Q9=97 AND Q10=1)

HAS HAD PRE-SERVICE TRAINING ONLY ☐ ↓
(Q9=00 OR GREATER AND Q10=2)
<u>THEN ANSWER 28a ONLY</u>

HAS NOT HAD <u>ANY</u> PRE OR IN SERVICE TRAINING ☐ → **Q29a**
(Q9=97 AND Q10=2)

TOPICS		Q28a. Did your **pre-service** training cover TOPIC?	Q28b. Have you ever attended an **in-service** training on TOPIC?	Q28c. What year was your most recent **in-service** training on TOPIC?	Q28d. Which organization or government ministry conducted this training?
(01)	Contraceptive technology update	YES . . . . .1<br>NO . . . . 2<br>DK…………..8	YES . . . . .1<br>NO . . . . .2 →**(02)**	[___|___|___|___]<br>**DK=9998**	______________<br>|___|___|
(02)	Exclusive breastfeeding counseling/LAM	YES . . . . .1<br>NO . . . . 2<br>DK…………..8	YES . . . . .1<br>NO . . . . .2 →**(03)**	[___|___|___|___]<br>**DK=9998**	______________<br>|___|___|
(03)	Natural family planning (rhythm method, etc.)	YES . . . . .1<br>NO . . . . 2<br>DK…………..8	YES . . . . .1<br>NO . . . . .2 →**(04)**	[___|___|___|___]<br>**DK=9998**	______________<br>|___|___|
(04)	SDM/Cycle beads	YES . . . .1<br>NO . . . .2<br>DK…………..8	YES . . . ….… .1<br>NO . . . . . …....2 →**(05)**	[___|___|___|___]<br>**DK=9998**	______________<br>|___|___|
(05)	Emergency Contraceptive	YES . . . . .1<br>NO . . . . 2<br>DK…………..8	YES . . . . .1<br>NO . . . . .2 →**(06)**	[___|___|___|___]<br>**DK=9998**	______________<br>|___|___|
(06)	Oral pills	YES . . . . .1<br>NO . . . . 2<br>DK…………..8	YES . . . . .1<br>NO . . . . .2 →**(07)**	[___|___|___|___]<br>**DK=9998**	______________<br>|___|___|
(07)	FP counseling skills	YES . . . . .1<br>NO . . . . 2<br>DK…………..8	YES . . . . .1<br>NO . . . . .2 →**(08)**	[___|___|___|___]<br>**DK=9998**	______________<br>|___|___|
(08)	Clinical skills on IUD	YES . . . . .1<br>NO . . . . 2<br>DK…………..8	YES . . . . .1<br>NO . . . . .2 →**(09)**	[___|___|___|___]<br>**DK=9998**	______________<br>|___|___|
(09)	Clinical skills on injectable contraceptive	YES . . . . .1<br>NO . . . . 2<br>DK…………..8	YES . . . . .1<br>NO . . . . .2 →**(10)**	[___|___|___|___]<br>**DK=9998**	______________<br>|___|___|
(10)	Clinical skills on implant	YES . . . . .1<br>NO . . . . 2<br>DK…………..8	YES . . . . .1<br>NO . . . . .2 →**(11)**	[___|___|___|___]<br>**DK=9998**	______________<br>|___|___|
(11)	Clinical skills on Female Sterilization	YES . . . . .1<br>NO . . . . 2<br>DK…………..8	YES . . . . .1<br>NO . . . . .2 →**(12)**	[___|___|___|___]<br>**DK=9998**	______________<br>|___|___|
(12)	Clinical skills on male sterilization	YES . . . . .1<br>NO . . . . 2<br>DK…………..8	YES . . . . .1<br>NO . . . . .2 →**(13)**	[___|___|___|___]<br>**DK=9998**	______________<br>|___|___|
(13)	Management of incomplete abortion (Post-Abortion Care)	YES . . . . .1<br>NO . . . . 2<br>DK…………..8	YES . . . . .1<br>NO . . . . .2 →**(14)**	[___|___|___|___]<br>**DK=9998**	______________<br>|___|___|
(14)	Manual vacuum aspiration (MVA)	YES . . . . .1<br>NO . . . . 2<br>DK…………..8	YES. . . ... . .1<br>NO . . . . .2→**(Q29a)**	[___|___|___|___]<br>**DK=9998**	______________<br>|___|___|

QUESTIONNAIRE IDENTIFICATION NO: [__|__|__|__|__|__|__|__]
5 digit facility code + 3 digit prov code

Now I would like to ask you some questions about your knowledge and provision of various methods of family planning. If you have provided a particular method before, we are also interested in the availability and quality of the materials required to provide that method.

METHOD	29a. Can you please tell me which of the following best describes your knowledge of [METHOD]:<br>1. You know METHOD sufficiently well to counsel and provide/assist in provision to a client;<br>2. You know METHOD sufficiently well to counsel, but not to provide;<br>3. You know little about METHOD and would not feel comfortable counseling or providing;<br>8. You do not know METHOD at all	29b. Have you provided (assisted with) [METHOD] to clients at this facility?	29c. Have you experienced any stockouts in this facility that lasted more than 24 hours of [METHOD] in the last one year?	29d. If yes, how many total days of stockouts did this facility have in the last ONE YEAR of [METHOD] (all stockouts combined)?	29e. Have you experienced a lack of essential equipment needed to provide [METHOD] in the last ONE YEAR?	29f. If Yes, how many total days did you lack essential equipment needed to provide [METHOD] in the last ONE YEAR?
(01) Combined oral pill	PROVIDE & COUNSEL…………….1<br>COUNSEL, NOT PROVIDE…..2<br>KNOW LITTLE ABOUT……….3 } **(02)**<br>DO NOT KNOW……………….8	YES……..1<br>NO………2 → **(02)**	YES………...1<br>NO…………2 → **(02)**<br>PRESCRIPTION ONLY………..3 → **(02)**	DAYS… [ ][ ][ ]<br>CONSTANT PROBLEM…995<br>DON'T KNOW..998		
(02) Progestin-only pill	PROVIDE & COUNSEL…………….1<br>COUNSEL, NOT PROVIDE…..2<br>KNOW LITTLE ABOUT……….3 } **(03)**<br>DO NOT KNOW……………….8	YES……..1<br>NO………2 → **(03)**	YES………...1<br>NO…………2 → **(03)**<br>PRESCRIPTION ONLY………..3 → **(03)**	DAYS… [ ][ ][ ]<br>CONSTANT PROBLEM…995<br>DON'T KNOW..998		
(03) Injectables	PROVIDE & COUNSEL…………….1<br>COUNSEL, NOT PROVIDE…..2<br>KNOW LITTLE ABOUT……….3 } **(04)**<br>DO NOT KNOW……………….8	YES……..1<br>NO………2 → **(04)**	YES………...1<br>NO…………2 → **(04)**<br>PRESCRIPTION ONLY……….3 → **(04)**	DAYS… [ ][ ][ ]<br>CONSTANT PROBLEM…995<br>DON'T KNOW..998		

Now I would like to ask you some questions about your knowledge and provision of various methods of family planning. If you have provided a particular method before, we are also interested in the availability and quality of the materials required to provide that method.

METHOD	29a. Can you please tell me which of the following best describes your knowledge of [METHOD]:<br>1. You know METHOD sufficiently well to counsel and provide/assist in provision to a client;<br>2. You know METHOD sufficiently well to counsel, but not to provide;<br>3. You know little about METHOD and would not feel comfortable counseling or providing;<br>8. You do not know METHOD at all	29b. Have you provided (assisted with) [METHOD] to clients at this facility?	29c. Have you experienced any stockouts in this facility that lasted more than 24 hours of [METHOD] in the last one year?	29d. If yes, how many total days of stockouts did this facility have in the last ONE YEAR of [METHOD] (all stockouts combined)?	29e. Have you experienced a lack of essential equipment needed to provide [METHOD] in the last ONE YEAR?	29f. If Yes, how many total days did you lack essential equipment needed to provide [METHOD] in the last ONE YEAR?
(04) Male condom	PROVIDE & COUNSEL……………1<br>COUNSEL, NOT PROVIDE…..2<br>KNOW LITTLE ABOUT……….3<br>DO NOT KNOW………………8<br>(2, 3, 8 → **(05)**)	YES……..1<br>NO………2 → **(05)**	YES………..1<br>NO…………2 → **(05)**<br>PRESCRIPTION ONLY……….3 → **(05)**	DAYS… [__][__][__]<br>CONSTANT PROBLEM…995<br>DON'T KNOW..998		
(05) Female condom	PROVIDE & COUNSEL……………1<br>COUNSEL, NOT PROVIDE…..2<br>KNOW LITTLE ABOUT……….3<br>DO NOT KNOW………………8<br>(2, 3, 8 → **(06)**)	YES……..1<br>NO………2 → **(06)**	YES………..1<br>NO…………2 → **(06)**<br>PRESCRIPTION ONLY……….3 → **(06)**	DAYS… [__][__][__]<br>CONSTANT PROBLEM…995<br>DON'T KNOW..998		
(06) Emergency contraception	PROVIDE & COUNSEL……………1<br>COUNSEL, NOT PROVIDE…..2<br>KNOW LITTLE ABOUT……….3<br>DO NOT KNOW………………8<br>(2, 3, 8 → **(07)**)	YES……..1<br>NO………2 → **(07)**	YES………..1<br>NO…………2 → **(07)**<br>PRESCRIPTION ONLY……….3 → **(07)**	DAYS… [__][__][__]<br>CONSTANT PROBLEM…995<br>DON'T KNOW..998		

Now I would like to ask you some questions about your knowledge and provision of various methods of family planning. If you have provided a particular method before, we are also interested in the availability and quality of the materials required to provide that method.

METHOD	29a. Can you please tell me which of the following best describes your knowledge of [METHOD]:<br>1. You know METHOD sufficiently well to counsel and provide/assist in provision to a client;<br>2. You know METHOD sufficiently well to counsel, but not to provide;<br>3. You know little about METHOD and would not feel comfortable counseling or providing;<br>8. You do not know METHOD at all	29b. Have you provided (assisted with) [METHOD] to clients at this facility?	29c. Have you experienced any stockouts in this facility that lasted more than 24 hours of [METHOD] in the last one year?	29d. If yes, how many total days of stockouts did this facility have in the last ONE YEAR of [METHOD] (all stockouts combined)?	29e. Have you experienced a lack of essential equipment needed to provide [METHOD] in the last ONE YEAR?	29f. If Yes, how many total days did you lack essential equipment needed to provide [METHOD] in the last ONE YEAR?
(07) Spermicide	PROVIDE & COUNSEL……………1<br>COUNSEL, NOT PROVIDE…..2<br>KNOW LITTLE ABOUT……….3<br>DO NOT KNOW………………8<br>(2, 3, 8) → **(08)**	YES……..1<br>NO………2 → **(08)**	YES………...1<br>NO…….……2 → **(08)**<br>PRESCRIPTION ONLY………..3 → **(08)**	DAYS… [__][__][__]<br>CONSTANT PROBLEM…995<br>DON'T KNOW..998		
(08) Diaphragm	PROVIDE & COUNSEL……………1<br>COUNSEL, NOT PROVIDE…..2<br>KNOW LITTLE ABOUT……….3<br>DO NOT KNOW………………8<br>(2, 3, 8) → **(09)**	YES……..1<br>NO………2 → **(09)**	YES………..1<br>NO…………2 → **(09)**<br>PRESCRIPTION ONLY……….3 → **(09)**	DAYS… [__][__][__]<br>CONSTANT PROBLEM…995<br>DON'T KNOW..998		
(09) SDM/Cycle beads	PROVIDE & COUNSEL……………1<br>COUNSEL, NOT PROVIDE…..2<br>KNOW LITTLE ABOUT……….3<br>DO NOT KNOW………………8<br>(2, 3, 8) → **(10)**	YES……..1<br>NO………2 → **(10)**	YES………..1<br>NO…………2 → **(10)**<br>PRESCRIPTION ONLY……….3 → **(10)**	DAYS… [__][__][__]<br>CONSTANT PROBLEM…995<br>DON'T KNOW..998		

**Now I would like to ask you some questions about your knowledge and provision of various methods of family planning. If you have provided a particular method before, we are also interested in the availability and quality of the materials required to provide that method.**						
METHOD	29a. Can you please tell me which of the following best describes your knowledge of [METHOD]:<br>1. You know METHOD sufficiently well to counsel and provide/assist in provision to a client;<br>2. You know METHOD sufficiently well to counsel, but not to provide;<br>3. You know little about METHOD and would not feel comfortable counseling or providing;<br>8. You do not know METHOD at all	29b. Have you provided (assisted with) [METHOD] to clients at this facility?	29c. Have you experienced any stockouts in this facility that lasted more than 24 hours of [METHOD] in the last one year?	29d. If yes, how many total days of stockouts did this facility have in the last ONE YEAR of [METHOD] (all stockouts combined)?	29e. Have you experienced a lack of essential equipment needed to provide [METHOD] in the last ONE YEAR?	29f. If Yes, how many total days did you lack essential equipment needed to provide [METHOD] in the last ONE YEAR?
(10) IUD	PROVIDE & COUNSEL…………….1<br>COUNSEL, NOT PROVIDE…..2<br>KNOW LITTLE ABOUT……….3<br>DO NOT KNOW……………….8<br>(2, 3, 8 → **(11)**)	YES……..1<br>NO………2→ **(11)**	YES………...1<br>NO…….……2 → **(29e)**<br>PRESCRIPTION ONLY……….3 →**(11)**	DAYS…<br>[ ][ ][ ]<br>CONSTANT PROBLEM…995<br>DON'T KNOW..998	YES……..1<br>NO………2→ **(11)**	DAYS…<br>[ ][ ][ ]<br>CONSTANT PROBLEM…995<br>DK…...998
(11) Implants	PROVIDE & COUNSEL…………….1<br>COUNSEL, NOT PROVIDE…..2<br>KNOW LITTLE ABOUT……….3<br>DO NOT KNOW……………….8<br>(2, 3, 8 → **(12)**)	YES……..1<br>NO………2→ **(12)**	YES………...1<br>NO…….……2 → **(29e)**<br>PRESCRIPTION ONLY……….3 →**(12)**	DAYS…<br>[ ][ ][ ]<br>CONSTANT PROBLEM…995<br>DON'T KNOW..998	YES……..1<br>NO………2→ **(12)**	DAYS…<br>[ ][ ][ ]<br>CONSTANT PROBLEM…995<br>DK…...998
(12) Female sterilization	PROVIDE & COUNSEL…………….1<br>COUNSEL, NOT PROVIDE…..2<br>KNOW LITTLE ABOUT……….3<br>DO NOT KNOW……………….8<br>(2, 3, 8 → **(13)**)	YES……..1<br>NO………2→ **(13)**			YES……..1<br>NO………2→ **(13)**	DAYS…<br>[ ][ ][ ]<br>CONSTANT PROBLEM…995<br>DK…...998
(13) Male sterilization	PROVIDE & COUNSEL…………….1<br>COUNSEL, NOT PROVIDE…..2<br>KNOW LITTLE ABOUT……….3<br>DO NOT KNOW……………….8<br>(2, 3, 8 → **(14)**)	YES……..1<br>NO………2→ **(14)**			YES……..1<br>NO………2→ **(14)**	DAYS…<br>[ ][ ][ ]<br>CONSTANT PROBLEM…995<br>DK…...998

QUESTIONNAIRE IDENTIFICATION NO:[__|__|__|__|__|__|__|__]
5 digit facility code + 3 digit prov code

	29a. Can you please tell me which of the following best describes your knowledge of [METHOD]:<br>1. Know the method sufficiently well to counsel and recommend to client<br>2. Know little about the method and would not feel comfortable counseling or recommending<br>8. Do not know method	29b. Have you ever recommended [METHOD] to clients at this facility?
(14) Natural methods (Rhythm, periodic abstinence, withdrawal)	COUNSEL & RECOMMEND……………..….1<br>KNOW LITTLE ABOUT……………..2 → (15)<br>DO NOT KNOW……………………...8 → (15)	Yes……..1<br>No………2
(15) Exclusive breastfeeding method (LAM)	COUNSEL & RECOMMEND……………..….1<br>KNOW LITTLE ABOUT……………..2 → Q30<br>DO NOT KNOW……………………..8 → Q30	Yes……..1<br>No………2

Q30. CHECK Q29A:

PROVIDES AND/OR COUNSELS ANY FP METHOD (ANY Q29A(1-13) = 1 OR 2; Q29A(14-15)=1) ☐ ↓

DOES NOT PROVIDE AND DOES NOT COUNSEL ANY FP METHOD (ALL Q29A(1-13) = 3 OR 8 AND Q29A (14-15)= 2 OR 8) ☐ → **Q36**

**Now I would like to ask you specifically about the contraceptive methods that you provide. (ASK ONLY ABOUT THE FAMILY PLANNING METHODS THE RESPONDENT IS PROVIDING – Q29b)**						
METHOD	Q31a. What is the minimum age that you would offer this [METHOD]?	Q31b. What is the maximum age that you would offer this [METHOD]?	Q31c. Is there a minimum number of children a person must have before you will offer [METHOD]?	Q31d. What is that minimum number of children?	Q31e. Do you require a partner's consent before you will provide [METHOD]?	Q31f. Would you offer METHOD to an unmarried person?
(1) Combined oral pills	[__|__]<br>NO MIN…...93<br>DK……….98	[__|__]<br>NO MAX…….93<br>DK……….98	YES . . .1<br>NO . . ...2 →**Q31e**<br>DK…….8→**Q31e**	[__|__]	YES . . . .1<br>NO 2	YES . . . .1<br>NO 2
(2) Progestin-only pill	[__|__]<br>NO MIN…...93<br>DK……….98	[__|__]<br>NO MAX…….93<br>DK……….98	YES . . .1<br>NO . . ...2 →**Q31e**<br>DK…….8→**Q31e**	[__|__]	YES . . . .1<br>NO 2	YES . . . .1<br>NO 2
(3) Injectables	[__|__]<br>NO MIN…...93<br>DK……….98	[__|__]<br>NO MAX……..93<br>DK……….98	YES . . .1<br>NO . . ...2 →**Q31e**<br>DK…….8→**Q31e**	[__|__]	YES . . . .1<br>NO 2	YES . . . .1<br>NO 2
(4) Male condom	[__|__]<br>NO MIN…...93<br>DK……….98	[__|__]<br>NO MAX……..93<br>DK……….98	YES . . .1<br>NO . . ...2 →**Q31e**<br>DK…….8→**Q31e**	[__|__]	YES . . . .1<br>NO 2	YES . . . .1<br>NO 2
(5) Female condom	[__|__]<br>NO MIN…...93<br>DK……….98	[__|__]<br>NO MAX……..93<br>DK……….98	YES . . .1<br>NO . . ...2 →**Q31e**<br>DK…….8→**Q31e**	[__|__]	YES . . . .1<br>NO 2	YES . . . .1<br>NO 2
(6) Emergency contraceptive	[__|__]<br>NO MIN…...93<br>DK……….98	[__|__]<br>NO MAX………93<br>DK……….98	YES . . .1<br>NO . . ...2 →**Q31e**<br>DK…….8→**Q31e**	[__|__]	YES . . . .1<br>NO 2	YES . . . .1<br>NO 2
(7) Spermicide	[__|__]<br>NO MIN…...93<br>DK……….98	[__|__]<br>NO MAX…….93<br>DK……….98	YES . . .1<br>NO . . ...2 →**Q31e**<br>DK…….8→**Q31e**	[__|__]	YES . . . .1<br>NO 2	YES . . . .1<br>NO 2

(8) Diaphragm	[ ][ ]<br>NO MIN…...93<br>DK……….98	[ ][ ]<br>NO MAX…….93<br>DK……….98	YES . . .1<br>NO . . …2 →**Q30e**<br>DK…….8→**Q30e**	[ ][ ]	YES . . . .1<br>NO 2	YES . . . .1<br>NO 2
(9) SDM/Cycle beads	[ ][ ]<br>NO MIN…...93<br>DK……….98	[ ][ ]<br>NO MAX…….93<br>DK……….98	YES . . .1<br>NO . . …2 →**Q31e**<br>DK…….8→**Q31e**	[ ][ ]	YES . . . .1<br>NO 2	YES . . . .1<br>NO 2
(10) IUD	[ ][ ]<br>NO MIN…...93<br>DK……….98	[ ][ ]<br>NO MAX……..93<br>DK……….98	YES . . .1<br>NO . . …2 →**Q31e**<br>DK…….8→**Q31e**	[ ][ ]	YES . . . .1<br>NO 2	YES . . . .1<br>NO 2
(11) Implants	[ ][ ]<br>NO MIN…...93<br>DK……….98	[ ][ ]<br>NO MAX...93<br>DK……….98	YES . . .1<br>NO . . …2 →**Q31e**<br>DK…….8→**Q31e**	[ ][ ]	YES . . . .1<br>NO 2	YES . . . .1<br>NO 2
(12) Female sterilization	[ ][ ]<br>NO MIN…….93<br>DK……….98	[ ][ ]<br>NO MAX...93<br>DK……….98	YES . . .1<br>NO . . …2 →**Q31e**<br>DK…….8→**Q31e**	[ ][ ]	YES . . . .1<br>NO 2	YES . . . .1<br>NO 2
(13) Male sterilization	[ ][ ]<br>NO MIN….93<br>DK……….98	[ ][ ]<br>NO MAX...93<br>DK……….98	YES . . .1<br>NO . . …2 →**Q31e**<br>DK…….8→**Q31e**	[ ][ ]	YES . . . .1<br>NO 2	YES . . . .1<br>NO 2

Q32.	What do you do/tell the client when talking about FP to clients?<br><br>PROBE – Anything else?<br>**MULTIPLE RESPONSES POSSIBLE.**<br>**CIRCLE ALL MENTIONED.**	IDENTIFY REPRODUCTIVE GOALS OF CLIENT……A<br>PROVIDE INFORMATION ABOUT DIFFERENT FP METHODS……………………………………...……….B<br>DISCUSS THE CLIENT'S FP PREFERENCES……....C<br>HELP CLIENT SELECT A SUITABLE METHOD…..…D<br>EXPLAIN THE WAY TO USE THE SELECTED METHOD……………………..……………………..….E<br>EXPLAIN THE SIDE-EFFECTS………………………..F<br>EXPLAIN SPECIFIC MEDICAL REASONS TO RETURN……………………………….....……………..G<br>REQUEST FOR PARTNER'S CONSENT……………..H<br><br>OTHERS___________________________________ X<br>(SPECIFY)	
Q33.	**CHECK Q29B:**<br><br>PROVIDES HORMONAL METHODS (PILL OF ANY TYPE, INJECTABLE, IUD, OR IMPLANTS: Q29B(1)=1 OR Q29B(2)=1 OR Q29B(3)=1 OR Q29B(10)=1 OR Q29B(11)=1) [ ] ↓	DOES NOT PROVIDE HORMONAL METHODS (ALL OF THE FOLLOWING EQUAL "2" OR ARE SKIPPED: Q29B(1), Q29B(2), Q29B(3), Q29B(10), Q29B(11)) [ ] →	**Q35**
Q34.	What do you do for a new client who wants the pill or another hormonal method but is not having her menses?<br><br>**DO NOT READ OPTIONS**<br><br>PROBE WITH "Anything else?"<br><br>**MULTIPLE RESPONSES POSSIBLE.**<br>**CIRCLE ALL MENTIONED.**	QUESTION TO EXCLUDE PREGNANCY………….A<br>EXAMINE TO EXCLUDE PREGNANCY……………B<br>TEST TO EXCLUDE PREGNANCY…………………C<br>TELL HER TO COME BACK AT NEXT MENSES….D<br>TRY TO INDUCE MENSES…………………………..E<br>SUPPLY CONDOMS UNTIL NEXT MENSES………F<br>SUPPLY HORMONAL METHOD IF REASONABLY CERTAIN SHE IS NOT PREGNANT…………..…G<br>SUPPLY HORMONAL METHOD AND CONDOMS, ASK HER TO USE CONDOMS UNTIL NEXT MENSES…………………………………..……..…H<br>JUST GIVE HORMONAL METHOD……………..……. J<br>REQUEST FOR PARTNER'S CONSENT………….K<br>OTHER________________________________ X<br>(SPECIFY)	

Q35.	Which kind of personal and financial records do you complete each time you provide a client with family planning services?<br><br>**MULTIPLE RESPONSES POSSIBLE.**<br>**CIRCLE ALL MENTIONED.**	A CLIENT RECORD CARD/FORM…………………...A<br>AN ENTRY IN THE FP REGISTER…......................B<br>AN ENTRY IN THE FACILITY LOGBOOK/ REGISTER……………………………….………..C<br>INFORMAL NOTES IN A NOTEBOOK……………...D<br>A PAYMENT RECEIPT IF A FEE IS INVOLVED…..E<br>OTHER ________________________________X<br>(SPECIFY)<br>NO RECORD KEPT…………………………………...Y	

**INTEGRATION OF FAMILY PLANNING WITH OTHER SERVICES**			
Q36.	Which are the other services that you yourself provide to clients at this health facility? READ THE OPTIONS.<br><br>**MULTIPLE RESPONSES POSSIBLE.**<br>**CIRCLE ALL MENTIONED.**	ANTE-NATAL CARE………………………..……………A<br>DELIVERY SERVICES…………………………………..B<br>POST-NATAL CARE………...C<br>POST-ABORTION CARE……………………………….D<br>CHILD IMMUNIZATION…………………………………E<br>CHILD GROWTH MONITORING……………………….F<br>OTHER CURATIVE SERVICES FOR WOMEN………G<br>OTHER CURATIVE SERVICES FOR CHILDREN…..H<br>HIV/AIDS MANAGEMENT………………………………I<br>PMTCT……………………………………………………..J<br>VCT………………………………………………………...K<br>NONE OF THESE………………………………………..Y →	**Q89**
Q37.	**CHECK Q36:**<br><br>IF OPTION A (ANTENATAL CARE) IS CIRCLED ☐ ↓	IF OPTION A (ANTENATAL CARE) IS **NOT** CIRCLED ☐ →	**Q46**
Q38.	During Antenatal care, do you provide information about FP routinely?	YES...1<br>NO...2 →	**Q44**

Q39.	What do you do/tell the client when talking about FP during antenatal care?<br><br>PROBE: "ANYTHING ELSE?"<br>**MULTIPLE RESPONSES POSSIBLE.**<br>**CIRCLE ALL MENTIONED.**	HELP THE WOMAN SELECT A SUITABLE METHOD FOR POST-DELIVERY…………………………….……..A<br>INFORM ABOUT THE IMPORTANCE OF USING FP BY 40 DAYS POSTPARTUM…………………………..…B<br>PROVIDE INFORMATION ON LAM……………………….C<br>EXPLAIN SIDE-EFFECTS……………………………..….D<br>ENCOURAGE WOMEN TO WAIT FOR SOME TIME BEFORE THE NEXT PREGNANCY………….…………E<br>REQUEST FOR PARTNER'S CONSENT…………..……...F<br>OTHERS: ____________________________________ X<br>(SPECIFY)	
Q40.	Do you tell women where they can obtain an FP method after delivery?	YES...1<br>NO...2	
Q41.	Do you use counseling job aids to provide FP services during antenatal care?	YES...1<br>NO...2 →	**Q46**
Q42.	Which job aids do you use?<br><br>PROBE: "ANYTHING ELSE?"<br>**MULTIPLE RESPONSES POSSIBLE.**<br>**CIRCLE ALL MENTIONED.**	NATIONAL STANDARD OF PRACTICE (SOP) FOR FP SERVICES ………………………………………….A<br>FMOH FP SERVICES PERFORMANCE STANDARDS……………………………………….……….B<br>WHO MEDICAL ELIGIBILITY CRITERIA (MERC)….….…C<br>NURHI FP COUNSELING FLIP CHARTS…………………D<br>GATHER CHART……………………………………………..E<br>FP METHOD CHART (WALL TYPE) ………………………F<br>NURHI SMS FP COMMODITY TRACKING JOB AID ……G<br>OJT MANUALS (THREE COURSES)……………………….H<br>OTHER ___________________________________X<br>(SPECIFY)	

Q43.	Who provided these job aids?<br><br>**MULTIPLE RESPONSES POSSIBLE. CIRCLE ALL MENTIONED.**	NURHI/FPPN ………………………... ..A<br>UNFPA ………………………………….B<br>WHO …………………………………….C<br>SFH ………………………………………….D<br>JHPIEGO ……………………………….E<br>MARIE STOPES ………………………F<br>PATH ………………………………….G<br>PPFN …………………………………….H<br>STATE/ MOH ……………………………I<br>MCHIP ………………………………….J<br>TSHIP…………………………………..K<br>UNICEF…………………………………L<br>OTHER ____________________________ X<br><br>DON"T KNOW………………………Z	**ALLL SKIP TO Q46**
Q44.	Why are you not able to provide FP information routinely during antenatal care visits?<br><br>**MULTIPLE RESPONSES POSSIBLE. CIRCLE ALL MENTIONED.**	ADEQUATE CONTRACEPTIVE METHODS FREQUENTLY UNAVAILABLE……………………………………………..A<br>AVAILABLE CONTRACEPTIVES OFTEN PAST EXPIRATION DATE……………………………………...B<br>LACK OF STERILE EQUIPMENT SO NO POINT DISCUSSING…………………………………………...C<br>LACK OF FUNCTIONAL EQUIPMENT SO NO POINT DISCUSSING……………………………………………..D<br>NO INTEREST IN PROVIDING FP INFORMATION………..E<br>LACK KNOWLEDGE ABOUT FP……………………………..F<br>DO NOT FEEL ADEQUATELY TRAINED TO PROVIDE FP INFORMATION…………………………………………G<br>NO INTEREST IN FP ON THE PART OF THE PATIENTS…H<br>OVERLOAD OF WORK/NO TIME TO DISCUSS…………….I<br>NO NEED TO……………………………………………………K<br>NOT A PROFITABLE SERVICE TO PROVIDE……………..L<br><br>OTHERS________________________________X<br>(SPECIFY)	
Q45.	Would you be willing to include family planning information routinely in your antenatal care services/visits?	YES...1<br>NO..2	
Q46.	**CHECK Q36:**<br><br>IF OPTION B (DELIVERY CARE) IS CIRCLED ☐ ↓	IF OPTION B (DELIVERY CARE) IS **NOT** CIRCLED ☐ →	**Q53**
Q47.	During delivery care (anytime before they are discharged from your facility), do you provide information about FP routinely?	YES...1<br>NO..2 →	**Q51**
Q48.	What do you do/tell the client when talking about FP during delivery care?<br><br>PROBE: "ANYTHING ELSE?"<br>**MULTIPLE RESPONSES POSSIBLE. CIRCLE ALL MENTIONED.**	HELP SELECT SUITABLE FP METHOD BY 40 DAYS POSTPARTUM……………………………….A<br>PROVIDE INFORMATION ON LAM……………………..…B<br>EXPLAIN SIDE-EFFECTS…………………………………C<br>EXPLAIN SPECIFIC MEDICAL REASONS TO RETURN…………………………………………………D<br>ENCOURAGE WOMEN TO WAIT SOME TIME BEFORE THE NEXT PREGNANCY……..…………..E<br>REQUEST FOR PARTNER'S CONSENT………………..F<br>OTHER_____________________________ X<br>(SPECIFY)	
Q49.	Do you tell women where they can obtain an FP method during delivery care?	YES...1<br>NO..2	
Q50.	Do you counsel women on LAM during delivery care?	YES...1<br>NO.. 2 →	**All skip to Q53**

Q51.	Why are you not able to provide FP information routinely during delivery care?<br><br>PROBE: "ANYTHING ELSE?"<br>**MULTIPLE RESPONSES POSSIBLE. CIRCLE ALL MENTIONED.**	ADEQUATE CONTRACEPTIVE METHODS FREQUENTLY UNAVAILABLE……A<br>AVAILABLE CONTRACEPTIVES OFTEN PAST EXPIRATION DATE……B<br>LACK OF STERILE EQUIPMENT SO NO POINT DISCUSSING……C<br>LACK OF FUNCTIONAL EQUIPMENT SO NO POINT DISCUSSING……D<br>NO INTEREST IN PROVIDING FP INFORMATION……E<br>LACK KNOWLEDGE ABOUT FP……F<br>DO NOT FEEL ADEQUATELY TRAINED TO PROVIDE FP INFORMATION……G<br>NO INTEREST IN FP ON THE PART OF THE PATIENTS…H<br>OVERLOAD OF WORK/NO TIME TO DISCUSS……I<br>NO NEED TO……K<br>NOT A PROFITABLE SERVICE TO PROVIDE……L<br><br>OTHERS________________X<br>(SPECIFY)	
Q52.	Would you be willing to include family planning information routinely in your delivery care services?	YES……1<br>NO……2	
Q53.	**CHECK Q36:**<br>IF OPTION C (POST-NATAL CARE) IS CIRCLED ☐ ↓	IF OPTION C (POST-NATAL CARE) IS **NOT** CIRCLED ☐ →	**Q62**
Q54.	During post-natal care visits, do you provide information about FP routinely?	YES……1<br>NO……2 →	**Q60**
Q55.	What do you do/tell the client when talking about FP during post-natal care visits?<br><br>PROBE: "ANYTHING ELSE?"<br>**MULTIPLE RESPONSES POSSIBLE. CIRCLE ALL MENTIONED.**	HELP SELECT SUITABLE FP METHOD BY 40 DAYS POSTPARTUM……A<br>PROVIDE INFORMATION ON LAM……B<br>EXPLAIN SIDE-EFFECTS……C<br>EXPLAIN SPECIFIC MEDICAL REASONS TO RETURN……D<br>ENCOURAGE WOMEN TO WAIT SOME TIME BEFORE THE NEXT PREGNANCY……E<br>REQUEST FOR PARTNER'S CONSENT……F<br>OTHER________________ X<br>(SPECIFY)	
Q56.	Do you tell women where they can obtain an FP method during post-natal care visits?	YES……1<br>NO……2	
Q57.	Do you use counseling job aids to provide FP services during postnatal care visits?	YES……1<br>NO……2 →	**Q62**
Q58.	Which job aids do you use?<br><br>PROBE: "ANYTHING ELSE?"<br>**MULTIPLE RESPONSES POSSIBLE. CIRCLE ALL MENTIONED.**	NATIONAL STANDARD OF PRACTICE (SOP) FOR FP SERVICES ……A<br>FMOH FP SERVICES PERFORMANCE STANDARDS……B<br>WHO MEDICAL ELIGIBILITY CRITERIA (MERC)……C<br>NURHI FP COUNSELING FLIP CHARTS……D<br>GATHER CHART……E<br>FP METHOD CHART (WALL TYPE) ……F<br>NURHI SMS FP COMMODITY TRACKING JOB AID ……G<br>OJT MANUALS (THREE COURSES)……H<br>NURHI MOBILE APP/CD……I<br>OTHER ________________X<br>(SPECIFY)	
Q59.	Who provided these job aids?	NURHI …… A<br>MOBILE DL SIM ……B<br>TSHIP……C<br>UNICEF……D<br>SMOH……E<br>SFH……F<br>OTHER ____________X<br>(SPECIFY)<br>DON'T KNOW ……Z	**ALL SKIP TO Q62**

Q60.	Why are you not able to provide FP information routinely during post-natal care visits?<br><br>PROBE: "ANYTHING ELSE?"<br>**MULTIPLE RESPONSES POSSIBLE.**<br>**CIRCLE ALL MENTIONED.**	ADEQUATE CONTRACEPTIVE METHODS FREQUENTLY UNAVAILABLE……………………………………………A<br>AVAILABLE CONTRACEPTIVES OFTEN PAST EXPIRATION DATE……………………………………B<br>LACK OF STERILE EQUIPMENT SO NO POINT DISCUSSING…………………………………………C<br>LACK OF FUNCTIONAL EQUIPMENT SO NO POINT DISCUSSING…………………………………………D<br>NO INTEREST IN PROVIDING FP INFORMATION………..E<br>LACK KNOWLEDGE ABOUT FP……………………………F<br>DO NOT FEEL ADEQUATELY TRAINED TO PROVIDE FP INFORMATION…………………………………………G<br>NO INTEREST IN FP ON THE PART OF THE PATIENTS…H<br>OVERLOAD OF WORK/NO TIME TO DISCUSS……………..I<br>NO NEED TO…………………………………………………K<br>NOT A PROFITABLE SERVICE TO PROVIDE………………L<br><br>OTHERS______________________________________X<br>(SPECIFY)	
Q61.	Would you be willing to include family planning information routinely in your postnatal care services?	YES...1<br>NO...2	
Q62.	**CHECK Q36:**<br><br>IF OPTION D (POST-ABORTION CARE) IS CIRCLED ☐ ↓	IF OPTION D (POST-ABORTION CARE) IS **NOT** CIRCLED ☐ →	**Q71**
Q63.	During a post abortion care, do you provide information about FP routinely?	YES..1<br>NO…………………………………………..2 →	**Q69**
Q64.	What do/tell the client when talking about FP during post abortion care visits?<br><br>PROBE: "ANYTHING ELSE?"<br>**MULTIPLE RESPONSES POSSIBLE.**<br>**CIRCLE ALL MENTIONED.**	IDENTIFY REPRODUCTIVE GOALS OF WOMAN...A<br>PROVIDE INFORMATION ABOUT DIFFERENT FP METHODS………………………………………B<br>DISCUSS THE CLIENT'S FP PREFERENCES……..C<br>HELP WOMEN SELECT A SUITABLE METHOD…..D<br>EDUCATE WOMEN TO USE THE SELECTED METHOD……………………………………………E<br>INFORM ABOUT HOW SOON AFTER ABORTION SHE MAY BECOME PREGNANT IF NOT USING CONTRACEPTION…………………………………...F<br>EXPLAIN SIDE-EFFECTS…………………………….…G<br>EXPLAIN SPECIFIC MEDICAL REASONS TO RETURN…………………………………………….…H<br>REQUEST FOR PARTNER'S CONSENT…………….I<br>OTHERS: _________________________________ X<br>(SPECIFY)	
Q65.	Do you tell women where they can obtain an FP method during post abortion care visits?	YES...1<br>NO...2	
Q66.	Do you use counseling job aids to provide FP services during post abortion care visits?	YES...1<br>NO...2 →	**Q71**
Q67.	Which job aids do you use?<br><br>PROBE: "ANYTHING ELSE?"<br>**MULTIPLE RESPONSES POSSIBLE.**<br>**CIRCLE ALL MENTIONED.**	NATIONAL STANDARD OF PRACTICE (SOP) FOR FP SERVICES ………………………………….A<br>FMOH FP SERVICES PERFORMANCE STANDARDS…………………………………………B<br>WHO MEDICAL ELIGIBILITY CRITERIA (MERC)…C<br>NURHI FP COUNSELING FLIP CHARTS…………D<br>GATHER CHART……………………………………..E<br>FP METHOD CHART (WALL TYPE) ………………F<br>NURHI SMS FP COMMODITY TRACKING JOB AID …………………………………………………….G<br>OJT MANUALS (THREE COURSES)…..………….H<br>OTHER _________________________________X<br>(SPECIFY)	

Q68.	Who provided these job aids?	NURHI …………………………………….. A<br>MOBILE DL SIM ……………………………..B<br>TSHIP………………………………………..C<br>UNICEF……………………………………..D<br>SMOH………………………………………..E<br>SFH…………………………………………..F<br>OTHER ________________________X<br>(SPECIFY)<br>DON'T KNOW ………………………………Z	**ALL SKIP TO Q71**
Q69.	Why are you not able to provide FP information routinely during post abortion care visits?<br><br>PROBE: "ANYTHING ELSE?"<br>**MULTIPLE RESPONSES POSSIBLE.**<br>**CIRCLE ALL MENTIONED.**	ADEQUATE CONTRACEPTIVE METHODS FREQUENTLY UNAVAILABLE………………………A<br>AVAILABLE CONTRACEPTIVES OFTEN PAST EXPIRATION DATE……………………..………B<br>LACK OF STERILE EQUIPMENT SO NO POINT DISCUSSING……………………………….....C<br>LACK OF FUNCTIONAL EQUIPMENT SO NO POINT DISCUSSING……………………......D<br>NO INTEREST IN PROVIDING FP INFORMATION…………………………………..E<br>LACK KNOWLEDGE ABOUT FP…………………....F<br>DO NOT FEEL ADEQUATELY TRAINED TO PROVIDE FP INFORMATION………………………..G<br>NO INTEREST IN FP ON THE PART OF THE PATIENTS…………………………………………H<br>OVERLOAD OF WORK/NO TIME TO DISCUSS…...I<br>NO NEED TO……………………………………………K<br>NOT A PROFITABLE SERVICE TO PROVIDE..……L<br>OTHERS______________________________X<br>(SPECIFY)	
Q70.	Would you be willing to include family planning information routinely in your post abortion care services/visits?	YES...1<br>NO..2	
Q71.	**CHECK Q36:**<br><br>IF **EITHER** OPTION E (CHILD IMMUNIZATION) OR OPTION F (CHILD GROWTH MONITORING) IS CIRCLED ☐ ↓	IF **NEITHER** OPTION E (CHILD IMMUNIZATION) **NOR** OPTION F (CHILD GROWTH MONITORING) IS CIRCLED ☐ →	**Q77**
Q72.	During child immunization/child growth monitoring, do you provide information about FP routinely?	YES...1<br>NO……………………………………………….2 →	**Q75**
Q73.	What do you do/tell clients when talking about FP during child immunization or child growth monitoring visits?<br><br>PROBE: "ANYTHING ELSE?"<br>**MULTIPLE RESPONSES POSSIBLE.**<br>**CIRCLE ALL MENTIONED.**	IDENTIFY REPRODUCTIVE GOALS OF WOMAN...A<br>PROVIDE INFORMATION ABOUT DIFFERENT FP METHODS………………………………………B<br>DISCUSS THE CLIENT'S FP PREFERENCES……..C<br>HELP WOMEN SELECT A SUITABLE METHOD…..D<br>EDUCATE WOMEN TO USE THE SELECTED METHOD…………………………………….…..E<br>EXPLAIN SIDE-EFFECTS…………………….……F<br>EXPLAIN SPECIFIC MEDICAL REASONS TO RETURN……………………………………...…G<br>REQUEST FOR PARTNER'S CONSENT……………H<br>OTHERS: ______________________________ X<br>(SPECIFY)	
Q74.	Do you tell women where they can obtain an FP method?	Yes...1<br>No..2	**All skip to Q77**

Q75.	Why are you not able to provide FP information routinely?<br><br>PROBE: "ANYTHING ELSE?"<br>**MULTIPLE RESPONSES POSSIBLE.**<br>**CIRCLE ALL MENTIONED.**	ADEQUATE CONTRACEPTIVE METHODS FREQUENTLY UNAVAILABLE……… ……..A<br>AVAILABLE CONTRACEPTIVES OFTEN PAST EXPIRATION DATE……………………… …..B<br>LACK OF STERILE EQUIPMENT SO NO POINT DISCUSSING……………………………………..C<br>LACK OF FUNCTIONAL EQUIPMENT SO NO POINT DISCUSSING……………………….D<br>NO INTEREST IN PROVIDING FP INFORMATION…………………………………….E<br>LACK KNOWLEDGE ABOUT FP…………………….F<br>DO NOT FEEL ADEQUATELY TRAINED TO PROVIDE FP INFORMATION…………………..G<br>NO INTEREST IN FP ON THE PART OF THE PATIENTS…………………………………….....H<br>OVERLOAD OF WORK/NO TIME……………….....I<br>NO NEED TO…………………………………………K<br>NOT A PROFITABLE SERVICE TO PROVIDE…….L<br><br>OTHERS_______________________________X<br>(SPECIFY)	
Q76.	Would you be willing to include family planning information routinely in your child immunization or child growth monitoring visits?	YES...1<br>NO...2	
Q77.	**CHECK Q36**:<br><br>IF **EITHER** OPTION G (CURATIVE SERVICES FOR WOMEN) OR H (CURATIVE SERVICES FOR CHILDREN) IS CIRCLED ☐ ↓	IF **NEITHER** OPTION G (CURATIVE SERVICES FOR WOMEN) **NOR** H (CURATIVE SERVICES FOR CHILDREN) IS CIRCLED ☐ →	**Q83**
Q78.	While providing curative services to women or children, do you provide information on FP routinely?	YES...1<br>NO...2 →	**Q81**
Q79.	What are the main activities you follow when talking about FP to clients?<br><br>PROBE: "ANYTHING ELSE?"<br>**MULTIPLE RESPONSES POSSIBLE.**<br>**CIRCLE ALL MENTIONED.**	IDENTIFY REPRODUCTIVE GOALS OF WOMAN..A<br>PROVIDE INFORMATION ABOUT DIFFERENT FP METHODS……………..…………….…….B<br>DISCUSS THE CLIENT'S FP PREFERENCES….C<br>HELP WOMEN SELECT A SUITABLE METHOD…………………………….…………...D<br>EDUCATE WOMEN TO USE THE SELECTED METHOD……………………….……………...….E<br>EXPLAIN SIDE-EFFECTS…………………………F<br>EXPLAIN SPECIFIC MEDICAL REASONS TO RETURN…………………………………...….G<br>OTHERS: ____________________________ X<br>(SPECIFY)	
Q80.	Do you tell women where they can obtain an FP method?	YES...1<br>NO...2 } →	**All skip to Q83**
Q81.	Why are you not able to provide FP information routinely?<br><br>PROBE: "ANYTHING ELSE?"<br>**MULTIPLE RESPONSES POSSIBLE.**<br>**CIRCLE ALL MENTIONED.**	ADEQUATE CONTRACEPTIVE METHODS FREQUENTLY UNAVAILABLE……… ……..A<br>AVAILABLE CONTRACEPTIVES OFTEN PAST EXPIRATION DATE……………………… …..B<br>LACK OF STERILE EQUIPMENT SO NO POINT DISCUSSING……………………………………..C<br>LACK OF FUNCTIONAL EQUIPMENT SO NO POINT DISCUSSING……………………….D<br>NO INTEREST IN PROVIDING FP INFORMATION…………………………………….E<br>LACK KNOWLEDGE ABOUT FP…………………….F<br>DO NOT FEEL ADEQUATELY TRAINED TO PROVIDE FP INFORMATION…………………..G<br>NO INTEREST IN FP ON THE PART OF THE PATIENTS…………………………………….....H<br>OVERLOAD OF WORK/NO TIME……………….....I<br>NO NEED TO…………………………………………K<br>NOT A PROFITABLE SERVICE TO PROVIDE…….L<br><br>OTHERS_______________________________X<br>(SPECIFY)	

Q82.	Would you be willing to include family planning information routinely in your curative care services/visits for women or children?	YES.....1<br>NO.....2	
Q83.	**CHECK Q36:**<br><br>IF **ANY** OF THE OPTIONS I (HIV/AIDS MANAGEMENT), OPTION J (PMTCT), OR K (VCT) ARE CIRCLED ☐ ↓	IF **NONE** OF THE OPTIONS I (HIV/AIDS MANAGEMENT), OPTION J (PMTCT), OR K (VCT) ARE CIRCLED ☐ →	**Q89**
Q84.	While providing HIV-related services (HIV/AIDS management, PMTCT, and/or VCT) to women and men, do you provide information on FP routinely?	YES.....1<br>NO.....2 →	**Q87**

Q85.	What are the main activities you follow when talking about FP to clients?<br><br>PROBE: "ANYTHING ELSE?"<br>**MULTIPLE RESPONSES POSSIBLE.**<br>**CIRCLE ALL MENTIONED.**	IDENTIFY REPRODUCTIVE GOALS OF WOMAN...A<br>PROVIDE INFORMATION ABOUT DIFFERENT FP METHODS.....B<br>DISCUSS THE CLIENT'S FP PREFERENCES........C<br>HELP WOMEN SELECT A SUITABLE METHOD.....D<br>EDUCATE WOMEN TO USE THE SELECTED METHOD.....E<br>EXPLAIN SIDE-EFFECTS.....F<br>EXPLAIN SPECIFIC MEDICAL REASONS TO RETURN.....G<br>DISCUSS HIV/AIDS PREVENTION METHODS......H<br>DISCUSS METHODS NOT RECOMMENDED FOR HIV POSITIVE (LAM, IUD).....I<br>RECOMMEND ALWAYS USE CONDOM IN ADDITION TO OTHER FP METHODS.....J<br>REQUEST FOR PARTNER'S CONSENT.....K<br>OTHERS: ______________ X<br>(SPECIFY)	
Q86.	Do you tell women where they can obtain an FP method?	YES.....1<br>NO.....2 } →	**All skip to Q89**
Q87.	Why are you not able to provide FP information routinely?<br><br>PROBE: "ANYTHING ELSE?"<br>**MULTIPLE RESPONSES POSSIBLE.**<br>**CIRCLE ALL MENTIONED.**<br>.	ADEQUATE CONTRACEPTIVE METHODS FREQUENTLY UNAVAILABLE.........A<br>AVAILABLE CONTRACEPTIVES OFTEN PAST EXPIRATION DATE.........B<br>LACK OF STERILE EQUIPMENT SO NO POINT DISCUSSING.....C<br>LACK OF FUNCTIONAL EQUIPMENT SO NO POINT DISCUSSING.....D<br>NO INTEREST IN PROVIDING FP INFORMATION.....E<br>LACK KNOWLEDGE ABOUT FP.....F<br>DO NOT FEEL ADEQUATELY TRAINED TO PROVIDE FP INFORMATION.....G<br>NO INTEREST IN FP ON THE PART OF THE PATIENTS.....H<br>OVERLOAD OF WORK/NO TIME.....I<br>NO NEED TO.....K<br>NOT A PROFITABLE SERVICE TO PROVIDE.......L<br><br>OTHERS______________X<br>(SPECIFY)	
Q88.	Would you be willing to include family planning information routinely in your HIV-related services/visits for women and men?	YES.....1<br>NO.....2	

**Integrated Supportive Supervision (ISS)**			
Q89.	Have you received an integrated supportive supervision visit in the last three months?	YES.....1<br>NO.....2 →	**Q93**
Q90.	Which organization/group visited you?	NURHI.....1<br>STATE GOVERNMENT.....2<br>FEDERAL GOVERNMENT.....3<br>OTHER ______________ 6<br>(SPECIFY)<br>DON'T KNOW..... 8	

Q91.	What type of feedback did you receive?	RECEIVED VERBAL/WRITTEN FEEDBACK..........A<br>RECEIVED HANDS ON/PRACTICAL TRAINING...B<br>RECEIVED DEMONSTRATION..............................C<br>OTHER________________________________X<br>(SPECIFY)<br><br>RECEIVED NO FEEDBACK....................................Y	**A-X SKIP TO Q93**
Q92.	Would you like to receive feedback?	YES...1<br>NO..2	
Q93.	Is this facility linked with another organization that provides family planning methods and materials at a discounted rate or for free (for example PPFN or SFH)?	YES..1<br>NO...2 →<br>DON'T KNOW...8 →	**Q94c**<br>**Q94c**

Q94a.	What is the name of the organization?	Q94b. What year did this facility begin to associate with each organization named?	
	1.	YEAR ……………... . . . [ ][ ][ ][ ]<br>DON'T KNOW 9998	
	2.	YEAR ……………... . . . [ ][ ][ ][ ]<br>DON'T KNOW 9998	
	3.	YEAR ……………... . . . [ ][ ][ ][ ]<br>DON'T KNOW 9998	
	4.	YEAR ……………... . . . [ ][ ][ ][ ]<br>DON'T KNOW 9998	
The following questions ask your perspective about the facility's administration. All of your responses will remain confidential and will not be shared with anyone outside the study team.			
Q94c	Is there one staff member in particular who advocates for family planning at this facility?	YES..1<br>NO..2 →<br><br>DON'T KNOW...8 →	**Q94e**<br><br>**Q94e**
Q94d	What cadre of staff are they?	OBSTETRICIAN/GYNECOLOGIST………..01<br>GENERAL SURGEON……………………………..……02<br>PEDIATRICIAN…………………………..….03<br>GENERAL PHYSICIAN……………………………….…..04<br>THEATRE NURSE…………………………………………05<br>NURSE/MIDWIFE…………………………..….06<br>NURSE……………………………………........07<br>MIDWIFE…………………………………..……08<br>COMMUNITY HEALTH EXTENSION WORKER (CHEW)………………………………………09<br>COMMUNITY HEALTH OFFICER (CHO)…………………………………………10<br>VCT COUNSELOR………………………………..11<br>OTHER ___________________________96<br>(SPECIFY)	

How supportive is this facility's administration of:					
		VERY UNSUPPORTIVE	UNSUPPORTIVE	SUPPORTIVE	VERY SUPPORTIVE
Q94e	Family Planning programs	1	2	3	4
Q94f	FP community outreach	1	2	3	4
Q94g	Training on FP methods	1	2	3	4
Q94h	Providing financial resources for FP efforts	1	2	3	4
Q94i	Ensuring minimal stock-outs of FP methods	1	2	3	4
Q94j	Partnering with other organizations to provide FP trainings in the facility	1	2	3	4
Q94k	Providing competitive wages for its employees	1	2	3	4
Q94l	Ensuring adequate staffing levels	1	2	3	4
Q94m	Minimizing staff turnover	1	2	3	4

Q95.	RECORD THE TIME<br><br>IN 24 HOUR FORMAT	HOUR ………… [ ][ ]	MINUTES …….. [ ][ ]

Thank you very much for taking the time to answer my questions. Once again, any information you have given will be kept completely confidential. Have a good day!
**COMMENTS:**
